# Supplementary material for: Development of a recombinase polymerase amplification lateral flow assay for the detection of active Trypanosoma evansi infections
Source: PLoS Negl Trop Dis. 2020 Feb 18;14(2):e0008044. doi: 10.1371/journal.pntd.0008044 (PMC7048301; doi:10.1371/journal.pntd.0008044)
Supplement: Attachment — Submitted filename: Review of Li 2019 PLoS NTD.docx [file pntd.0008044.s001.docx]

**Review of Li 2019 PLoS NTD**

**10/15/2019**

**Title:** Development of a Recombinase Polymerase Amplification Lateral Flow Assay for the Detection of Active Trypanosoma evansi Infections

**Authors:** Zheng Li1,2, Joar Esteban Pinto Torres1, Julie Goossens1, Benoit Stijlemans1,3, Yann G.-J. Sterckx2Y, Stefan Magez1,4,5Y*

**Summary**

This study performed initial characterization of an assay for detection of the animal pathogen *Trypanosoma evansi.* The assay detects a unique sequence in the genome, amplifying it by a recombinase polymerase reaction. The amplified fragments are detected by lateral flow with a signal line appearing when biotin coupled to one end of the amplicon binds to streptavidin coated test line and gold particle-anti-FAM binds to the FAM molecule incorporated at the other end of the amplicon. The system is optimized for incubation temperature and time of incubation. Minimum quantity of T. evansi DNA detectible and lack of reactivity with similar parasite species DNA are demonstrated. The assay’s ability to detect T. evansi infection in blood samples of infected mice was compared to end point PCR and microscopy.

**Comments**

The authors have assembled a test system for a disease of agricultural importance that is a particular problem in resource-limited areas of the world. The isothermal amplification and lateral flow detection are features that have potential to make the assay easier to perform in the field. The amount of characterization and validation performed is sufficient to consider this a proof of concept for the assay. As far as testing was done, the performance of the assay is good. At 100fg of DNA detected, the analytical limit is close to one parasite, which is good sensitivity. The authors have not tested clinical sensitivity in the sense of the lower limit of parasites in a blood sample from an infected animal. The infected animal tests are good, though limited in number and are taken from animals that would be expected to have a large parasite load. Further validation of the assay would require a larger number of animals over a range of infection conditions including lower parasite loads below the analytical limit of detection. Assaying infected animals after drug treatment is an important demonstration of the test’s ability to evaluate cure, a limitation of serological tests.

This study involves the use of research animals (mice). Research with animals should be under the guidance of an Institutional Animal Care and Use Committee. I do not see where this is a requirement of PLOS NTD, however, the authors have not mentioned approval of an IACUC for the work performed, which I believe should be required. If the authors did the animal work under such supervision, please include a statement to that effect in the manuscript.

**Minor issues that could be corrected**

1. On page 6, line 125, the statement, “described previously in 2.5 with the exception of the addition of 2.1 μl of both forward” is not clear. The number 2.5 may refer to an earlier section that was numbered in an earlier draft. Please remove the number an replace with a proper reference to the place.
2. The description of RPA and its illustration in Figure 1 are incomplete and don’t fully explain how amplification occurs. The reference cited, #62, Daher et al. 2016, and the figure contained in that reference are much clearer. The explanation that the primers repeatedly re-invade the amplicons to generate new copies of the amplicon and the arrow on the figure in Daher 2016 indicating isothermal cycles of invasion and extension would help make the point in Figure 1 of the manuscript under review.
3. There is no discussion of a cutoff criterion that distinguishes a positive result from a negative result in the lateral flow detection. For example, in Figure 5A, Day 3 lateral flow results, the band for mouse #2 is not much more visible that mouse #6 (negative control), yet mouse #2 is labeled positive. My observations were made on the Figure5.tif, zooming in for a close-up and out for an overall view. Some method for objective evaluation of the band must be developed. Other strip assays build in a low positive and a high positive. A test band must be darker than the low positive and the high positive is included in an algorithm to assign a numerical value to the band intensity.
4. On page 10, line 254, “the advantage of the TevRPA-LF is that it can be performed on crude genomic DNA samples.” A stronger statement could be made such as, “TevRPA-LF was effective when performed with crude genomic DNA.
