# Supplementary material for: Development of a recombinase polymerase amplification lateral flow assay for the detection of active Trypanosoma evansi infections
Source: PLoS Negl Trop Dis. 2020 Feb 18;14(2):e0008044. doi: 10.1371/journal.pntd.0008044 (PMC7048301; doi:10.1371/journal.pntd.0008044)
Supplement: Attachment — Submitted filename: Response_to_reviewers_comments.docx [file pntd.0008044.s002.docx]

**Reply to the referees** **(#PNTD-D-19-01398)**

**Development of a recombinase polymerase amplification lateral flow assay for the detection of active *Trypanosoma evansi* infections**

Zeng Li, Joar Pinto, Julie Goossens, Benoit Stijlemans, Yann G.-J. Sterckx, Stefan Magez

First and foremost, we would like to thank the reviewers for their critical assessment of the submitted manuscript and for their constructive feedback.

**RESPONSE TO REVIEWER 1**

**Comments related to summary and general comments**

Comment 1: ‘*This paper describes the development of a new diagnostic test for Trypanosoma evansi based on Recombinase Polymerase Amplification technology. The RPA test is an alternative to existing DNA-based PCR or LAMP diagnostic tests. The work is clearly explained on the whole. The limitations of the test in terms of sensitivity and ability to detect all T. evansi need to be included.*’

The limitation of the *Tev*RPA-LF described here with regards to the detection of only Type A *T. evansi* (and thus not Type B) was previously mentioned in the manuscript but could indeed have been stated more explicitly. To further clarify this limitation, we have made the following textual changes (changes highlighted in yellow)

- Abstract: ‘As an alternative, we developed a recombinase polymerase amplification (RPA) test for Type A *T. evansi*.’
- Introduction: ‘In this present study, we describe the development of the first recombinase polymerase amplification lateral flow assay for the detection of active Type A *T. evansi* infections (*Tev*RPA-LF)’
- Results and Discussion: ‘This is the case for a specific region (bp 1 to 1300) of the *T. evansi* RoTat1.2 VSG gene, which forms the target of the *Tev*RPA-LF for *T. evansi* detection. This limits the use of the TevRPA-LF described here to the detection of Type A *T. evansi*, and not Type B.’
- Conclusion: ‘In this paper, we have described the development of a *Tev*RPA-LF, a test that specifically detects active Type A *T. evansi* infections by amplifying a region in the *T. evansi* RoTat1.2 VSG gene.’

Comment 2: ‘*Abstract – the sensitivity of the RPA test could be mentioned.*’

We agree and now report the experimental sensitivity of the *Tev*RPA-LF in the abstract. This now reads as follows (changes/additions highlighted in yellow):

‘**Conclusions/Significance** Compared to other DNA-based parasite detection methods (such as PCR and LAMP), the *T. evansi* RPA-LF (*Tev*RPA-LF), described in this paper is an interesting alternative because of its simple read-out (user-friendly), short execution time (15 minutes), experimental sensitivity of 100 fg purified genomic *T. evansi* DNA, and ability to be carried out at a moderate, constant temperature (39°C). Therefore, the *Tev*RPA-LF is an interesting tool for the detection of active *T. evansi* infections.’

Comment 3: ‘*I believe that Type A and B T. evansi were named after their kDNA minicircle type and therefore the PCR diagnostic tests based on minicircles should be mentioned.*’

Following the reviewer’s suggestion, we made textual changes and have included an additional reference (changes/additions highlighted in yellow)

‘*T. evansi* parasites are classified into two groups based on their kDNA minicircle type [24], which are characterised by the presence (Type A) or absence (Type B) of the gene encoding the RoTat1.2 variant surface glycoprotein (VSG) [25,26].’

Ref [24] Masiga and Gibson (1990). Mol Biochem Parasitol vol. 40, pp. 279-283

Comment 4: ‘*On p3 line 49 – some explanation of how RPA works would be useful at this point for readers unfamiliar with the technique.*’

We agree with the reviewer’s suggestion and have moved the first reference to Figure 1 to the Introduction section as follows (changes/additions are highlighted in yellow).

‘An interesting alternative to PCR is the so-called Recombinase Polymerase Amplification (RPA) [48]. The reaction mechanism of RPA has been reviewed elsewhere [49,50] and is summarized in Fig 1 (the figure legend contains a detailed explanation of the RPA reaction).’

Comment 5: ‘*Table 1 – the host is given as “cattle” which is a plural term and out of line with the other host terms used.  Use cow instead.*’

We have changed the text accordingly.

Comment 6: ‘*Line 125 “in 2.5 with the exception of the addition of…”. Meaning needs to be clarified.’*

We have clarified this section of the text as follows (changes/additions highlighted in yellow)

‘Briefly, the RPA reaction was assembled as described above (Materials and Methods subsection 'Development and optimization of the *Tev*RPA assay') with the exception of…’

Comment 7: ‘*Line 144 and 221 - useful to give the equivalent number of parasites.’*

While it would indeed be interesting to give the equivalent number of parasites, we would prefer not to make any potential overstatement in this regard. Based on our calculations, 100 fg purified genomic DNA would correspond to 33 trypanosomes/ml. A report in the literature describes that 100 fg would correspond to 1 single parasite (Borst *et al*., 1982. Molecular and Biochemical Parasitology vol. 6, pp. 13-23) as was also mentioned by reviewer #2. These discrepancies probably depend on the efficiencies of the employed DNA extraction methods. Given the existing discrepancies, we would not want to present any erroneous statements.

Comment 8: ‘*Line 175 It should be pointed out the RoTat 1.2 gene is not present in all T. evansi – as in ref 27.’*

To address this comment, we would like to refer to our reply to Comment 1.

Comment 9: ‘*Line 184 Figure legend but no figure included – figures are at end with no list of figure legends to refer to.’*

The figures and figure legends were prepared according to the PLoS submission guidelines, which are the following ‘*figure captions in the manuscript text in read order, immediately following the paragraph where the figure is first cited. Do not include captions as part of the figure files or submit them in a separate document*.’

Comment 10: ‘*Ref 38 seems incomplete.’*

The missing information for this reference has been added.

Comment 11: ‘*Fig 1 is rather fussy and overcomplicated – it needs to be simplified, e.g. the trypanosome on the left is redundant, there is no need to show DNA as a double helix. Perhaps the figure could be split into RPA and RPA-LF?’*

As Reviewer #3 also provided some suggestions related to this figure, we have adapted Figure 1 by taking into account as many comments/suggestions as possible.

Comment 12: ‘*Fig 5 is it necessary to show every mouse result?’*

We appreciate the reviewer’s concern. We initially considered to move this figure to the supplemental data section of the manuscript. However, one of the authors strongly felt that the figure should be included in the paper’s main body. As the other reviewers have not made any comment with respect to this figure, we would propose to maintain this figure as a part of the main paper and thus not have it moved to the supplemental data.

**RESPONSE TO REVIEWER 2**

**Comments related to summary and general comments**

Comment 1: ‘*What is the added value of the test over T. evansi LAMP? Part of the authors published in 2018 in Vet Parasitol a study on RoTat 1.2 LAMP for sensitive T. evansi detection for diagnosis as well as test of cure (Tong et al. 2018). Why is this RPA needed and what is the added value over LAMP? LAMP amplification can be detected in real-time in simple closed-tube formats so what is the added value of a LF molecular test?*’

We acknowledge the fact that we and others have spent significant efforts in the past in the development of LAMP-based assays for African trypanosomosis. However, we feel it is always a benefit to have a back-up technology. Hence, while developing a new approach for the diagnosis of *T. evansi* trypanosomosis, we adopted a technology that has not been used before in the context of animal disease diagnosis. With LF being a very familiar format in field settings, we believe that this format can offer a valid alternative for LAMP in settings where the LAMP format is not considered to be favorable by the end user.

Comment 2: *‘The authors state that the test can be a valid alternative for the currently used screening tools. I’m not sure this is the case. How would the test fit in a diagnostic flow for surra given the already available tests RoTat 1.2 CATT, Surra Sero K-Set, T. evansi trypanolysis, PCR and LAMP? Where is the intended use of the tests? Currently Surra is rarely diagnosed in the field but at reference labs where molecular lab facilities are mostly available. This should be presented/discussed in the conclusions section.*’

We believe that the test we have developed here has several advantages over most of the existing test, in a relevant field setting, for the following reasons:

(1) CATT and the Sero K-test measure exposure to trypanosomes as the test is antibody-based. Taken the unpredictable half-life of a polyclonal antibody response to parasite exposure in a field setting, there are plenty of reasons why an antibody test could provide a false positive result. In settings where regular indiscriminate treatment is applied, antibody testing is also not the advised way for specific diagnosis. Finally, there are multiple diseases that induce polyclonal B cell activation (including trypanosomosis itself). In the presence of such infections, antibody measurements will also result in an increase in false positive scores. Finally, multiple reports have documented the occasional poor performance of antibody measurement in terms of specificity and sensitivity, so alternative methods appear to be very welcome.

- Tehseen *et al.* (2015). Parasitological, serological and molecular survey of *Trypanosoma evansi* infection in dromedary camels from Cholistan Desert, Pakistan. *Parasites & Vectors* vol. 8:415 (doi:10.1186/s13071-015-1002-3)
- Regassa et al. (2015). Trypanosome infection in dromedary camels in Eastern Ethiopia: Prevalence, relative performance of diagnostic tools and host related risk factors. *Veterinary Parasitology* vol. 211:175 (doi:10.1016/j.vetpar.2015.04.008)
- Njiru *et al.* (2004). Detection of *Trypanosoma evansi* in camels using PCR and CATT/*T. evansi* tests in Kenya. *Veterinary Parasitology* vol. 124:187 (doi:10.1016/j.vetpar.2004.06.029)

(2) While trypanolytic activity is a more specific measurement than CATT, a positive titer could be the result of a past infection or repeated exposure without successful infection, again because the test is antibody-based. In addition, this test requires the growth of *T. evansi* parasites and in case the test requires high specificity, it should actually be performed with a parasite that is relevant for the suspected ongoing infection, which is actually virtually impossible to guarantee if the mean question is whether an animal is infected or not. Finally, the test requires that experimental animals are sacrificed on a continuous basis in order to allow the test to be performed. Taken the principle of the 3Rs (reduce, refine and replace), any test that could reliably replace the lysis assay, should be chosen over the latter.
(3) PCR is a technique that is unsuitable to be executed in a field setting and for livestock diseases such as trypanosomosis there should be a drive towards the development of test that can be executed without the need of sample shipment to a specialized center.
(4) LAMP: as outlined above, we do agree that LAMP is a format that has its advantages in the field, as it detects the presence of parasites and can be executed with less technical restraints than PCR. But as argued above, the existence of a single format in our view is no argument to deny the development of an alternative method that might be preferred due the familiar format of the LF. RPA is not a technique that is widely applied in the field of animal diagnosis, hence, the application of the technique by itself already holds a specific value for the scientific community in our opinion. We do not see this as a LAMP versus RPA competition, but rather present a new technology to the field of trypanosomosis diagnosis.
(5) We agree that the existence of reference labs for Surra is important, but it is clear that the global spread of this infection brings challenges that need an improved response. Having a tool that can be used in the field will bring down the barriers that are now imposed by the need for sample shipment and off-site diagnosis. The development of a LF device for active-case detection in our view is a tool that can improve future diagnostic workflows, in particular if it is based on parasite detection.

Comment 3: *Lines 31-33 on the low PPV of CATT, do the authors have any data or reference to support this statement?*’

Since the goal of this paper is not to discuss the performance of CATT, we have removed the statement regarding the low PPV of CATT from the manuscript. The following references, however, present a discussion on the matter.

Cnops and Magez (2015). Chapter 17 in Human Emerging and Re-emerging infections. John Wiley & Sons, Ltd, pp. 319-335

AHAW *et al*. (2017). EFSA Journal vol. 15, e04892

Tehseen *et al.* (2015) Parasites & Vectors vol. 8, 415

Regassa *et al.* (2015). Veterinary Parasitology vol. 211, 175

Njiru *et al.* (2004). Veterinary Parasitology vol. 124, 187

Comment 4: *‘Data on analytical sensitivity of RPA in serial diluted T. evansi DNA: this should be complemented by applying conventional T. evansi PCR (table 2) on the same serial dilutions. Same for the mice experiments with and without Berenil treatment.’*

To answer the reviewer’s question, we have performed the *Tev*PCR on the same dilution series of *T. evansi* purified genomic DNA as for the *Tev*RPA-LF (Figure 3, panel C of the original manuscript). The result of this PCR is shown below.

Figure 1 – Additional experiment related to Figure 3.C of the original manuscript. Lanes 1-8, 10-fold dilution series of T. evansi purified genomic DNA starting at 10 ng μl−1 (1 μl was loaded onto the gel). Lane 1, 10 ng; Lane 2, 1 ng; Lane 3, 100 pg; Lane 4, 10 pg; Lane 5, 1 pg; Lane 6, 100 fg; Lane 7, 10 fg; Lane 8, 1 fg; Lane M, molecular mass marker; Lane N, negative control (no template DNA)

As expected, the *Tev*PCR performs better (1 fg DNA can be detected) than the RPA reaction (10 to 100 fg DNA can be detected), which can probably be directly correlated to the higher number of amplification cycles during *Tev*PCR compared to the RPA. We did not repeat any *in vivo* experiments as we do not expect that an RPA assay would outperform a PCR performed under optimal conditions. However, as indicated in the new version of the discussion section (lines 270-285), the premise of the paper was not to state that RPA would outperform a PCR carried out under controlled laboratory conditions, but that an RPA-LF based diagnostic test would be a viable alternative in terms of user-friendliness and field applicability.

Comment 5: *‘How do the authors avoid sample contamination with PCR products from previous runs when applying later flow post-amplification, especially since the authors present the test for field use?*’

We appreciate what is a very valid concern raised by the reviewer. However, such concerns apply to all molecular diagnostics tools employed in the field and come down to complying with good laboratory practices.

Comment 6: ‘*The study would be much stronger if a proof-of-concept can also be delivered on clinical samples.*’

The authors fully agree with this comment. Unfortunately, the opportunity to work with clinical samples did not present itself within the time frame in which this study was conducted. We are fully aware that testing the *Tev*RPA-LF on clinical samples would be the next step forward, which is why we have added the following statement in the Conclusion section of the manuscript (changes/additions highlighted in yellow).

‘Based on the above-mentioned findings, the newly developed *Tev*RPA-LF presented in this paper provides a proof-of-concept with the potential of becoming a valid alternative for currently used screening tools. Its further development will require an additional evaluation of its performance in both experimental and clinical animal infection models.’

**RESPONSE TO REVIEWER 3**

**Comments related to Methods**

Comment 1: ‘*There is no discussion of a cutoff criterion that distinguishes a positive result from a negative result in the lateral flow detection. For example, in Figure 5A, Day 3 lateral flow results, the band for mouse #2 is not much more visible than mouse #6 (negative control), yet mouse #2 is labeled positive. My observations were made on the Figure5.tif, zooming in for a close-up and out for an overall view. Some method for objective evaluation of the band must be developed. Other strip assays build in a low positive and a high positive. A test band must be darker than the low positive and the high positive is included in an algorithm to assign a numerical value to the band intensity. A numerical score is not required for the TevRPA-LF, but objective scoring is.*’

The reviewer makes a very valid point and we wholeheartedly agree. This is an issue that should definitely be included in the further evaluation of the *Tev*RPA-LF and is something we have not yet addressed at this stage of the development of the *Tev*RPA-LF.

Comment 2: ‘*This study involves the use of research animals (mice). Research with animals should be under the guidance of an Institutional Animal Care and Use Committee. I do not see where this is a requirement of PLOS NTD, however, the authors have not mentioned approval of an IACUC for the work performed, which I believe should be required. If the authors did the animal work under such supervision, please include a statement to that effect in the manuscript.*’

In Flanders, where the experiments have been performed, the ECAE is the equivalent of the IACUC mentioned by the reviewer. The Materials and Methods section starts with the following Ethics statement

‘All experiments, maintenance and care of the mice complied with the European Convention for the Protection of Vertebrate Animals (ECPVA) used for Experimental and Other Scientific Purposes guidelines (CETS n° 123) and were approved by the Ethical Committee for Animal Experiments (ECAE) at the Vrije Universiteit Brussel (Permit Number: 14-220-31).’

**Comments related to Results**

Comment 1: ‘*The results are clear and well presented other than the issue stated above regarding objective criteria for negative versus positive scoring of the Lateral Flow test band.*’

To address this comment, we would like to refer to our reply to Comment 1 related to the Methods section.

**Comments related to Conclusions**

Comment 1: ‘*The conclusion that the TevRPA-LF is a valid alternative for currently used screening tests overstates the evaluation that has been done. TevRPA-LF performed as well or better that alternatives in the few repetitions that were done. TevRPA-LF has promise to be easier to perform in the field than other available tests. However, the suggestion that it is ready to replace the other tests should come after more validation.*’

The authors fully agree with this comment, which is why we have added the following statement in the Conclusion section of the manuscript (changes/additions highlighted in yellow).

‘Based on the above-mentioned findings, the newly developed *Tev*RPA-LF presented in this paper provides a proof-of-concept with the potential of becoming a valid alternative for currently used screening tools. Its further development will require an additional evaluation of its performance in both experimental and clinical animal infection models.’

**Comments related to editorial and data presentation modifications**

Comment 1: ‘*On page 6, line 125, the statement, “described previously in 2.5 with the exception of the addition of 2.1 μl of both forward” is not clear. The number 2.5 may refer to an earlier section that was numbered in an earlier draft. Please remove the number and replace with a proper reference to the place the information was described.*’

We have clarified this section of the text as follows (changes/additions highlighted in yellow)

‘Briefly, the RPA reaction was assembled as described above (Materials and Methods subsection 'Development and optimization of the *Tev*RPA assay') with the exception of…’

Comment 2: ‘*The description of RPA and its illustration in Figure 1 are incomplete and don’t fully explain how amplification occurs. The reference cited, #62, Daher et al. 2016, and the figure contained in that reference are much clearer. The explanation that the primers repeatedly re-invade the amplicons to generate new copies of the amplicon and the arrow on the figure in Daher 2016 indicating isothermal cycles of invasion and extension would help make the point in Figure 1 of the manuscript under review.*’

We have generated an adapted version of Figure 1 following the reviewer’s suggestions.

Comment 3: ‘*On page 10, line 254, “the advantage of the TevRPA-LF is that it can be performed on crude genomic DNA samples.” A stronger statement could be made such as, “TevRPA-LF was effective when performed with crude genomic DNA" because this is in the results section. A “can be performed” statement would be appropriate in the Conclusion.*’

We have adapted the text according to the reviewer’s suggestions.

**Comments related to summary and general comments**

Comment 1: ‘*The authors have assembled a test system for a disease of agricultural importance that is a particular problem in resource-limited areas of the world. The isothermal amplification and lateral flow detection are features that have potential to make the assay easier to perform in the field. The amount of characterization and validation performed is sufficient to consider this a proof of concept for the assay. As far as testing was done, the performance of the assay is good. At 100fg of DNA detected, the analytical limit is close to one parasite, which is good sensitivity. The authors have not tested clinical sensitivity in the sense of the lower limit of parasites in a blood sample from an infected animal. The infected animal tests are good, though limited in number and are taken from animals that would be expected to have a large parasite load. Further validation of the assay would require a larger number of animals over a range of infection conditions including
lower parasite loads below the analytical limit of detection.  Assaying infected animals after drug treatment is an important demonstration of the test’s ability to evaluate cure, a limitation of serological tests.*’

We fully agree with reviewer’s suggestion. As mentioned above, we have adapted our statements in the Conclusion section according to the reviewer’s suggestions.
